# Decreased Cathepsin-K Mirrors the Severity of Subclinical Atherosclerosis in Kidney Transplant Recipients

**DOI:** 10.31083/j.rcm2309311

**Published:** 2022-09-13

**Authors:** Davide Bolignano, Marta Greco, Valentina Arcidiacono, Pierangela Presta, Alfredo Caglioti, Emilio Russo, Michele Andreucci, Omar Tripolino, Nazareno Carullo, Daniela Patrizia Foti, Giuseppe Coppolino

**Affiliations:** ^1^Renal Unit, “Magna Graecia'' University, 88100 Catanzaro, Italy; ^2^Clinical Pathology Lab, “Magna Graecia'' University, 88100 Catanzaro, Italy; ^3^Pharmacology Unit, “Magna Graecia” University, 88100 Catanzaro, Italy

**Keywords:** kidney transplantation, Cathepsin-K, carotid intima-media thickness, subclinical atherosclerosis

## Abstract

**Background:**

In kidney transplantation (Ktx) recipients, cardiovascular (CV) disease remains the leading cause of death. Abnormal carotid intima-media thickness (IMT) represents a valid indicator of incipient atherosclerosis also in this setting. Cathepsin-K (CatK) is a cysteine protease involved in vascular remodelling, as well as in progressive atherosclerosis. In this study we evaluated clinical predictors of CatK in Ktx recipients, with a particular focus on its possible relationships with subclinical atherosclerosis.

**Methods:**

Circulating CatK was measured in 40 stable Ktx recipients together with several laboratory, clinical and echocardiography parameters. 30 healthy subjects and 30 hemodialysis (HD) patients served as controls for CatK values. Carotid IMT was measured in Ktx and these subjects were then categorized according to age-gender reference cut-offs of normal IMT.

**Results:**

CatK levels were similar in Ktx recipients and healthy subjects but significantly reduced as compared to HD (*p* = 0.0001). In Ktx, at multivariate analyses CatK was associated with the LV end-diastolic volume (LVEDVi) (β = 0.514; *p* = 0.05), Ktx vintage (β = –0.333; *p* = 0.05) and mean IMT (β = –0.545; *p* = 0.05); this latter robust inverse association was confirmed also in another multivariate model with IMT as the dependent variable. Logistic regression analyses confirmed the beneficial meaning of CatK increase towards subclinical atherosclerosis [Odds Ratio (OR) 0.761; 95% Confidence Interval (CI) 0.569–0.918, *p* = 0.04]. At Receiver Operating Characteristics (ROC) analyses, CatK held a remarkable discriminatory power in identifying Ktx patients with abnormally increased IMT [Area Under the Curve (AUC) 0.763; 95% CI 0.601–0.926; *p* = 0.001]).

**Conclusions:**

In Ktx recipients, reduced CatK levels reflect the time-dependent improvement in the uremic milieu, cardiac adaptations and, above all, the severity of subclinical atherosclerosis. CatK measurement in Ktx may therefore hold significance for improving early CV risk stratification.

## 1. Introduction 

Patients with advanced chronic kidney disease (CKD) on hemodialysis (HD) treatment exhibits a remarkable risk of cardiovascular (CV) morbidity and mortality [1]. Although a successful kidney transplantation (Ktx) significantly improves such risk, the rate of CV events persists higher in Ktx recipients as compared with the general population and CV disease remains the leading cause of death, more so than infection or malignancy [2]. Increased carotid intima-media thickness (IMT) is largely considered as a footprint of incipient atherosclerosis, as well as a robust risk stratifier for CV mortality also in the Ktx setting [3]. Although Ktx often elicits a significant improvement in IMT as compared to patients remaining on maintenance dialysis [4], abnormally increased IMT persists in a large part of asymptomatic Ktx recipients and may worsen over time to frank atherosclerosis, driven by various factors such as immunosuppressive therapy, dyslipidaemia and diabetes [5, 6]. Cathepsin-K (CatK) is a cysteine protease endowed with collagenase and elastase activities, which has recently been implicated in the pathogenesis of progressive atherosclerosis [7]. High CatK mRNA/protein expression has been found in unstable atherosclerotic plaques [8] and CatK-deficiency was showed to protect atherosclerotic mice from disease progression [9]. Coronary artery disease (CAD) patients have altered circulating CatK levels; however, while some studies found direct associations between circulating CatK and the severity of CAD [10, 11], some others find opposite correlations, particularly in the presence of concomitant mineral bone disorders [12].

Recently, we reported increased CatK levels among chronic HD patients which reflected an altered bone mineral metabolism, also holding prognostic value for CV mortality [13, 14]. To the best of our knowledge, however, no study has analysed so far such protease in the context of Ktx, particularly in relationship with the presence of early, asymptomatic CV disease.

Starting from these premises, we thus aimed at conducting an exploratory study in a small cohort of Ktx recipients to evaluate clinical predictors of circulating CatK levels and its possible associations with subclinical atherosclerosis.

## 2. Materials and Methods

### 2.1 Patients’ Selection

Fifty-eight adult kidney transplant recipients (age >18) referred to the outpatient clinic of the University Hospital of Catanzaro, Italy, from November 2021 to February 2022 were screened to enter a pilot, observational, cross-sectional study. Cancer, infections, active inflammatory states, unstable renal function or severe renal impairment [Glomerular Filtration Rate (GFR) <15 mL/min/1.73 $m^{2}$, according to the CKD-Epi formula], peripheral vasculopathy and recent transplantation (<3 months) represented the main exclusion criteria. Main clinical, demographic and anthropometric parameters were recorded using a standardized, electronic case report form. The study was approved by the Local University Institutional Review Board and all participating subjects provided written informed consent.

### 2.2 Laboratory Measurements

Blood specimens were collected after an 8-hour overnight fast. Biochemical parameters and cardiac-specific biomarkers were measured in all patients by Cobas 8000 (Roche Diagnostics, Basel, Switerland) using the relative kits (Roche Diagnostics, Basel, Switzerland). Blood count analysis (Hb, RBC, WBC and platelet counts) was performed using ADVIA 2120i (Siemens Healthcare Diagnostics, Marburg, Germany). Fibrinogen was determined on BCS XP (Siemens, Healthcare Diagnostics, Marburg, Germany) using the Clauss method. All the above-mentioned assays were carried out according to the manufacturers’ instructions. Serum samples were centrifuged at 1227 g for 15 minutes at 4 °C and the aliquots stored at –80 °C until thawed for batch analysis. Cathepsin-K was measured in the blood using an ELISA commercially available kit (Novus Biological, Centennial, CO, US), according to the manufacturer’s instructions. The enzymatic reactions were quantified in an automatic microplate photometer. Measurements were made blind and in duplicate and levels were expressed as pg per mL. CatK was also measured in 30 healthy subjects and 30 patients undergoing chronic HD treatment who were matched with Ktx recipients for age and gender.

### 2.3 Cardiovascular Assessment

All Ktx recipients underwent a comprehensive cardiovascular assessment including blood pressure measurement at rest by a manual sphygmomanometer, a thorough echocardiographic examination and carotid echography for IMT evaluation. Echocardiography was performed using a GE Vivid E95 (General Electric Healthcare, Illinois, USA), with electrocardiographic monitoring during the exam. Left ventricular (LV) function was assessed by computing LV ejection fraction and the fractional shortening. In addition, the LV end-diastolic volume (LVEDVi) and a body-surface indexed LV mass (LVMi) were calculated, as indicated [15]. Right ventricular function was measured as the tricuspid annular plane systolic excursion (TAPSE) and Left and Right atrial volumes (RAVi, LAVi) were also measured as recommended [16].

Measurement of carotid IMT was performed in the posterior wall of both carotid arteries by mode B ultrasound with an ultrasonography device (LogiQ C5 premium, GE Medical Systems, China) equipped with a linear 8 cm probe operating at 8 MHz by an experienced operator. IMT was computed by measuring the thickness of the innermost two layers of intima-media, 5 mm before the bifurcation of the common carotid artery. Mean carotid IMT values were calculated as the average of absolute dx and sx measurements. Subclinical atherosclerosis was assumed in the presence of a mean carotid IMT value >0.9 mm and/or an unilateral IMT value over the 75th percentile of the established age- and sex-dependent reference ranges, in the absence of frank atherosclerotic plaques [17, 18, 19, 20].

### 2.4 Statistical Analysis

The statistical analysis was performed using the SPSS package (version 24.0; IBM corporation, Chicago, IL, USA), the MedCalc Statistical Software (version 14.8.1; MedCalc Software bvba, Ostend, Belgium) and the GraphPad prism package (version 8.4.2, GraphPad Software, San Diego, CA, USA). Data were presented as mean ± SD, median [IQ range] or frequency percentage as appropriate. Differences between groups were assessed by the unpaired *t*-test for normally distributed values, the Mann-Whitney U test for non-parametric values and the chi-square followed by a Fisher’s exact test for frequency distributions. The Pearson (R) and the Spearman (Rho) correlation coefficients were employed to test correlations between variables, as appropriate. Before testing correlations, all values showing a skewed distribution were log-transformed to better approximate normal distributions. Multiple regression analyses were performed by building two separate models including all univariate correlates of CatK and IMT values, respectively, in order to assess independent relationships. Data were expressed as partial correlation coefficients (β) and *p* value. Logistic regression analyses were performed to establish significant associations between the presence of subclinical atherosclerosis and any clinical variable which resulted different at baseline between the two study subgroups. To avoid co-linearity, average and absolute unilateral IMT values were excluded from the model. A Receiver Operating Characteristics (ROC) analysis was employed to calculate the area under the curve (AUC) and the best cut-off value for CatK considering the presence of subclinical atherosclerosis as status variable. All results were considered significant for *p* values ≤ 0.05.

## 3. Results

### 3.1 Main Characteristics of the Study Population

**Fig. 1.**
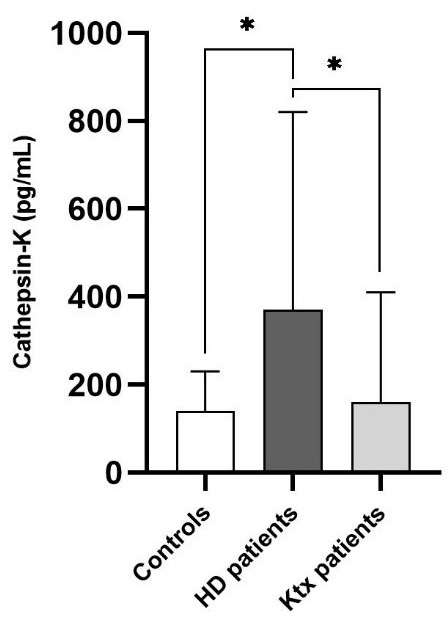
**Cathepsin-K levels in Ktx patients (n = 40) as compared with healthy matched controls (n = 30) and hemodialysis patients (n = 30)**. *p = 0.0001.

The final study population fulfilling the inclusion criteria consisted of 40 Ktx recipients. Mean age was 56.6 ± 12.5 years and 26 (65%) were male. Median transplantation vintage was 9 years (IQR 3–18) while the median dialysis duration before the transplant was 33.5 months (IQR 13–65). Most patients (85%) received a kidney from deceased donors. Only ten patients (25%) were diabetics while the majority (65%) were hypertensive under pharmacological control. Prevalence of other cardiovascular diseases was negligible. Combined immunosuppressive therapy included calcineurin inhibitors in 37 patients (92.5%), corticosteroids in 33 (82.5%), mycophenolate mofetil in 29 (72.5%) and m-TOR inhibitors in only 4 patients (10%). Nine patients (22.5%) were on treatment with statin/ezetimibe. Median serum creatinine was 1.33 mg/dL (IQR 1.10–2.11) with a median estimated GFR of 54.8 mL/min/$m^{2}$ (IQR 32.7–66.1). All patients had no or minimal proteinuria (median 0.15 g/24 h; IQR 0.10–0.40) and median PTH levels of 122.7 pg/mL (IQR 68.6–167.2). Serum calcium, phosphate, alkaline phosphatase and Vit-D were within the normal range in the majority of individuals, as well as the lipid and inflammatory profile. No relevant deviations from normal ranges were reported in the main echocardiography parameters. Mean carotid IMT values in the study cohort were 0.71 ± 0.25 mm. Only three patients (7.5%) showed values above 1.0 mm but none of them had overt evidence of atherosclerotic plaques. Median CatK levels were 160 [80–490] pg/mL. These latter values were apparently not different from those measured in healthy controls (140 [50–240] pg/mL; *p* = 0.20) but significantly lower as compared to matched individuals on maintenance HD (370 [210–1170] pg/mL; *p* = 0.0001; Fig. 1). Tables 1,2 summarize the main anthropometric, clinical, laboratory and cardiovascular parameters of the study population.

**Table 1.** ** Main anthropometric, clinical and laboratory parameters of the study population. Statistical differences between individuals with or without evidence of subclinical atherosclerosis (SubAth) are highlighted in bold**.

	All	SubAth	no-SubAth	*p*
	n = 40	n = 17	n = 23	
Age (yrs)	56.6 ± 12.5	57.6 ± 11.2	52.2 ± 13.5	0.20
Gender (%Male)	65	58.9	69.5	0.48
Dialysis vintage (mo.)	33.5 [13–65]	27 [14–58.5]	33.5 [10–79.5]	0.84
Ktx vintage (yrs)	9 [3–18]	14 [5.5–22]	4 [2–15.7]	0.10
DD Ktx (%)	85	88.2	82.6	0.66
BMI (${\mathrm{kg/m}}^{2}$)	26.1 ± 4.9	26.5 ± 6.1	25.6 ± 3.8	0.67
WHR (cm/cm)	0.92 ± 0.08	0.94 ± 0.09	0.91 ± 0.07	0.39
Current smokers (%)	15	17.6	13	0.69
**Diabetes (%)**	**25**	**41**	**13**	**0.04**
Coronary disease (%)	2.5	1.8	4.3	0.23
Heart failure (%)	5	5.9	8.7	0.21
Hypertension (%)	65	70.6	60.9	0.54
Immunosuppressive Therapy (%):				
*-Corticosteroids*	82.5	82.3	82.6	0.86
*-CNI*	92.5	94.1	91.3	0.81
*-MMF*	72.5	70.6	82.6	0.61
-m-TORi	10	11.7	8.6	0.74
Statin/Ezetimibe (%)	22.5	29.4	17.4	0.65
Glycemia (mg/dL)	96.5 ± 22.9	104.6 ± 29.4	90.6 ± 15	0.06
Serum creatinine (mg/dL)	1.33 [1.10–2.11]	1.21 [1.01–1.92]	1.38 [1.24–2.39]	0.10
eGFR (CKD-Epi mL/min/1.73 $m^{2}$)	54.8 [32.7–66.1]	60.9 [37.1–67.4]	53.7 [28.4–66.2]	0.31
Proteinuria (g/24 h)	0.15 [0.10–0.40]	0.14 [0.10–0.49]	0.16 [0.09–0.66]	0.82
Urea (mg/dL)	55 [43.5–89.5]	55 [45–75.5]	54 [42–91.2]	0.92
Uric Acid (mg/dL)	6.02 ± 1.41	6.07 ± 1.43	5.99 ± 1.46	0.86
Serum Phosphate (mg/dL)	3.34 ± 0.97	3.30 ± 1.12	3.36 ± 0.89	0.79
Serum Calcium (mg/dL)	9.6 ± 0.82	9.75 ± 0.87	9.63 ± 0.81	0.59
Parathormone (pg/mL)	122.7 [68.6–167.2]	121.8 [96.3–160.6]	117.8 [59.5–177.7]	0.97
**Alkaline Phosphatase (U/L)**	**80.4 ± 20.5**	**90.2 ± 19**	**74 ± 18.7**	**0.008**
Total Cholesterol (mg/dL)	184.6 ± 36.7	190.7 ± 39.3	177.2 ± 32.6	0.37
LDL Cholesterol (mg/dL)	109.5 ± 34.2	110.7 ± 33.5	105.3 ± 32.4	0.84
HDL Cholesterol (mg/dL)	58.1 ± 13.4	59.3 ± 13.9	56.9 ± 13.5	0.61
**Triglycerides (mg/dL)**	**140.5 ± 55.8**	**169.6 ± 63.4**	**120.6 ± 38.4**	**0.003**
Fibrinogen (mg/dL)	352.3 ± 99.1	363.5 ± 94.4	344.9 ± 106.1	0.54
25(OH)Vit-D (ng/mL)	27.1 ± 10.9	27.7 ± 10.1	26.7 ± 12.1	0.78
ESR (mm/h)	15 [9–27]	18 [9.5–26]	14.5 [9–28.5]	0.71
Albumin (g/dL)	4.38 ± 0.35	4.37 ± 0.29	4.39 ± 0.40	0.79
RBC (n × ${\mathrm{10}}^{6}$)	4.68 ± 0.82	4.72 ± 0.52	4.87 ± 1.17	0.80
Hb (g/dL)	12.6 ± 1.9	13.3 ± 1.9	12.1 ± 1.4	0.10
WBC (n × ${\mathrm{10}}^{3}$)	7.11 ± 1.97	6.08 ± 1.91	8.01 ± 2.26	0.11
PLT (n × ${\mathrm{10}}^{3}$)	217 ± 80.2	271.5 ± 131.2	214.2 ± 66.8	0.42
C-reactive protein (mg/L)	3.23 [2.13–4.10]	3.40 [2.13–4.8]	3.23 [2.13–3.42]	0.12
Ferritin (mg/dL)	38 [16.5–97]	39 [14.5–97]	42 [16.5–119.7]	0.71
TSAT (%)	31.7 ± 5.7	32.3 ± 5.2	31.2 ± 6.3	0.56
Serum iron (mg/dL)	66.1 ± 28.9	65.5 ± 22.6	67.4 ± 33.9	0.91
**Cathepsin-K (pg/mL)**	**160 [80–490]**	**260 [135–490]**	**140 [50–240]**	**0.002**

BMI, body mass index; CNI, calcineurin inhibitors; DD, deceased donor; eGFR, estimated glomerular filtration rate; ESR, erythrocyte sedimentation rate; Hb, haemoglobin; HDL, high density lipoprotein; Ktx, kidney transplantation; LDL, low density lipoprotein; MMF, mycophenolate mofetil; m-TORi, m-TOR inhibitors; PLT, platelets; RBC, red blood cells; TSAT, saturated transferrin; Vit-D, vitamin-D; WBC, white blood cells; WHR, waist-hip-ratio. Data are presented as mean (±SD), median [interquartile range] or percentage frequency.

**Table 2.** **Main cardiovascular and echocardiography parameters of the study population. Statistical differences between individuals with or without evidence of subclinical atherosclerosis (SubAth) are highlighted in bold**.

	All	SubAth	no-SubAth	*p*
	N = 40	n = 17	n = 23	
SBP (mmHg)	135 ± 18	136.5 ± 18.7	133.6 ± 18.2	0.66
DBP (mmHg)	85 ± 6.6	86.1 ± 7.6	84.3 ± 6	0.42
CK-MB (U/L)	1.80 [1.15–2.20]	1.6 [1.15–2.15]	1.85 [1–2.32]	0.57
Myoglobin (nmol/L)	41.5 [33–78.5]	41 [29–80.5]	43 [33.5–85.5]	0.37
hs-cTn (ng/L)	12.4 [7.3–24.6]	12.2 [6.7–19]	12.1 [7.5–26.9]	0.27
nt-pro-BNP (pg/mL)	220 [67–1266]	250 [85.5–411]	187.5 [65–1523]	0.92
**Mean carotid IMT (mm)**	**0.71 ± 0.25**	**0.85 ± 0.06**	**0.61 ± 0.13**	< **0.0001**
**Right carotid IMT (mm)**	**0.71 ± 0.32**	**0.90 ± 0.38**	**0.57 ± 0.17**	< **0.0001**
**Left carotid IMT (mm)**	**0.70 ± 0.22**	**0.89 ± 0.16**	**0.56 ± 0.14**	**<0.0001**
LAVi (${\mathrm{mL/m}}^{2}$)	35.1 ± 13	29.8 ± 10.6	31.2 ± 10.6	0.82
LVMi (${\mathrm{g/m}}^{2}$)	162 ± 54.3	161.5 ± 53.9	159.9 ± 60.4	0.97
LVEDVi (${\mathrm{mL/m}}^{2}$)	52.1 ± 6.8	48.4 ± 3.9	55.3 ± 7.4	0.09
Ejection Fraction (%)	59.3 ± 3.6	58.5 ± 6.9	57 ± 4.2	0.58
Vmax (m/s)	2.06 ± 0.54	1.92 ± 0.82	2.45 ± 0.07	0.22
TAPSE (mm)	21.8 ± 2.2	23.5 ± 0.7	20 ± 1.4	0.43
**E/e’**	**9.4 ± 3.9**	**7.1 ± 1.2**	**11.5 ± 2.1**	**0.04**
Fractional Shortening (%)	3.20 ± 0.59	3.35 ± 1.20	3.65 ± 1.34	0.75
RAVi (${\mathrm{mL/m}}^{2}$)	18.3 ± 7.3	14 ± 2.8	23 ± 8.4	0.40

SBP, systolic blood pressure; CK-MB, creatine-kinase MB; DBP, diastolic blood pressure; E/e’, early diastolic peak left ventricular inflow velocity (E)/early diastolic peak lateral mitral annular velocity (e’) ratio; hs-cTn, highly-sensitive c-troponin; IMT, carotid intima-media thickness; LAVi, left atrial volume index; LVEDVi, left-ventricular end diastolic volume index; LVMi, left ventricular mass index; nt-pro-BNP, n-terminal pro Brain Natriuretic Peptide; RAVi, right atrial volume index. TAPSE, tricuspid annular plane excursion; Vmax, peak aortic valve velocity. Data are presented as mean (±SD) or median [interquartile range].

### 3.2 Clinical Correlates of Cathepsin-K Levels in Ktx Recipients 

**Fig. 2.**
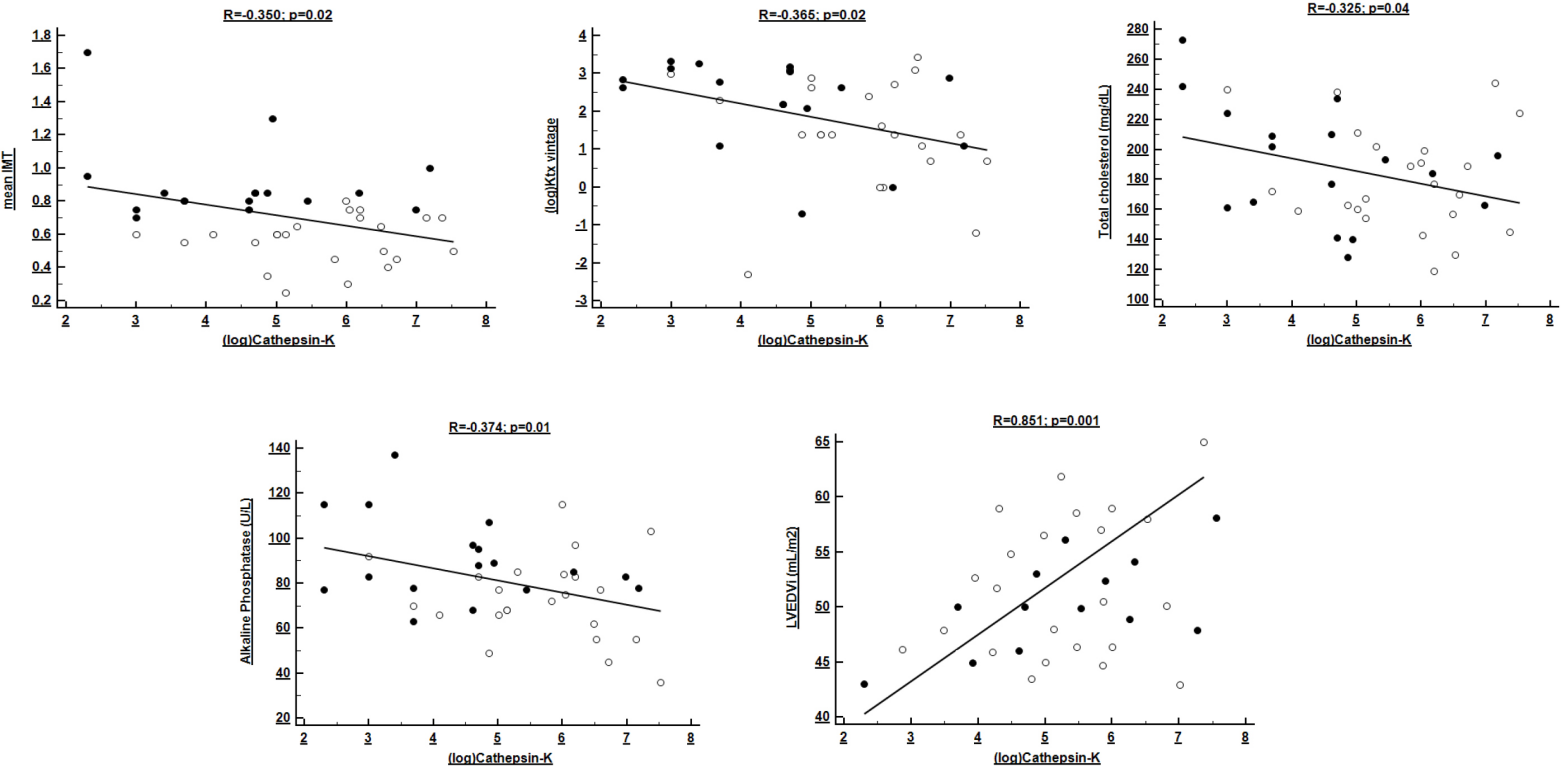
**Univariate correlates of (log transformed) Cathepsin-K levels in Ktx patients**. Black and white dots refer to individuals with or without subclinical atherosclerosis, respectively.

At univariate analyses, circulating CatK levels were inversely correlated with mean carotid IMT (R = –0.350; *p* = 0.02), Ktx vintage (R = –0.365; *p* = 0.02), total cholesterol (R = –0.325; *p* = 0.04) and alkaline phosphatase (R = –0.374; *p* = 0.01) while a direct association was found with left-ventricular end diastolic volume index (R = 0.851; *p* = 0.001). In a multivariate model including all univariate significant correlates, IMT (β = –0.545; *p* = 0.05), LVEDVi (β = 0.514; *p* = 0.05) and Ktx vintage (β = –0.333; *p* = 0.05) remained significant predictors of CatK values while the univariate correlations with alkaline phosphatase and cholesterol were lost. Of note this model resulted remarkably robust, explaining about 90% of the overall variation of CatK in this population (*p* = 0.01). Table 3 and Fig. 2 summarize clinical predictors of CatK.

**Table 3.** **Univariate and multiple regression analysis of (log-transformed) Cathepsin-K levels**.

	***Univariate correlation coefficient***	***p***
mean IMT	–0.350	0.02
(log)Ktx vintage	–0.365	0.02
Total cholesterol	–0.325	0.04
Alkaline phosphatase	–0.374	0.01
LVEDVi	0.851	0.001
	***Multivariate standardized correlation coefficient (β)***	***p***
**mean IMT**	**–0.545**	**0.05**
**LVEDVi**	**0.514**	**0.05**
**(log)Ktx vintage**	**–0.333**	**0.05**
Total cholesterol	–0.278	0.13
Alkaline phosphatase	0.278	0.26

Multiple R = 0.95, $R^{2}$ = 91%; *p* = 0.01.

### 3.3 Clinical Correlates of Carotid IMT

**Fig. 3.**
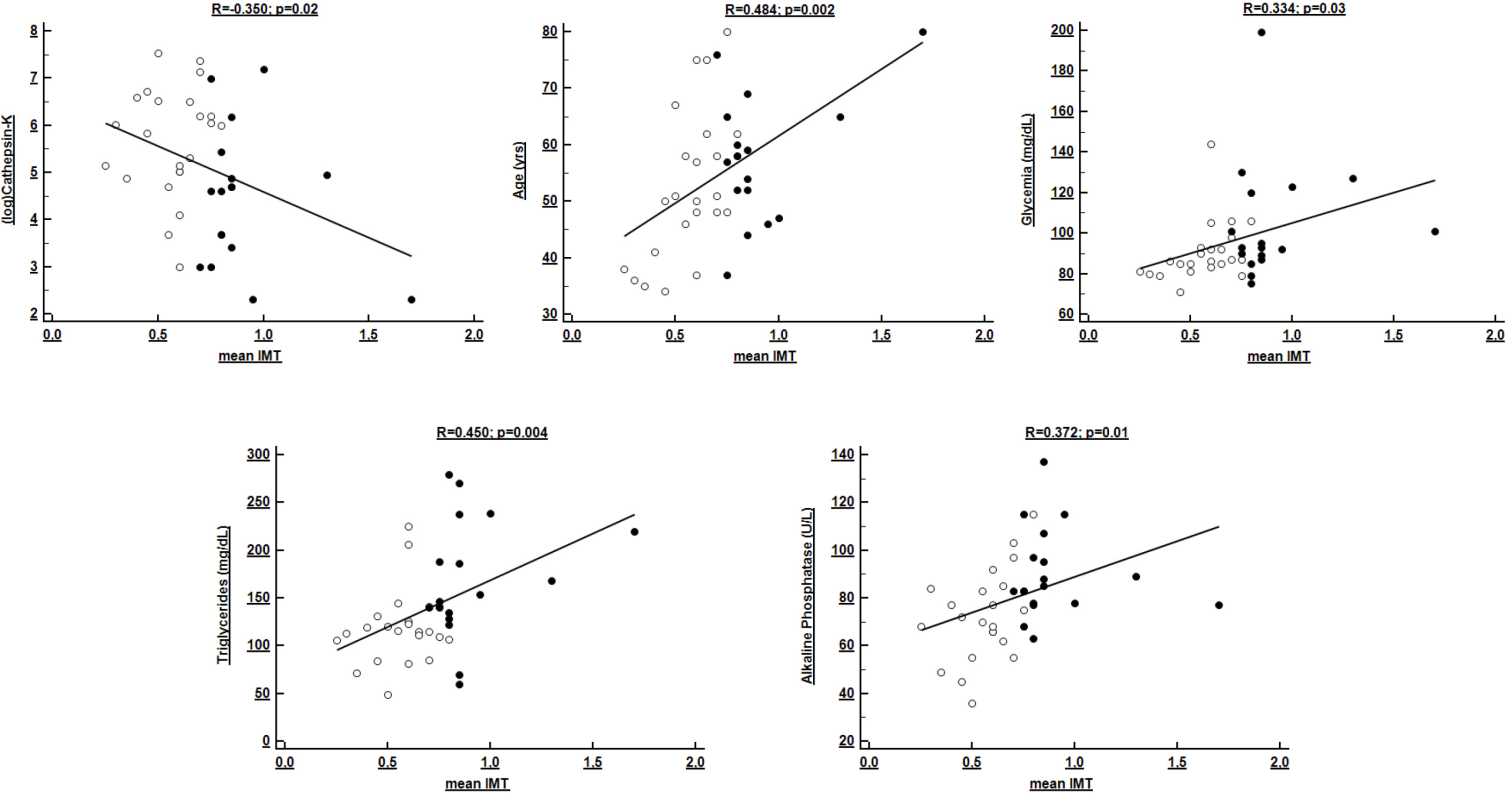
**Univariate correlates of mean carotid IMT in Ktx patients**. Black and white dots refer to individuals with or without subclinical atherosclerosis, respectively.

Carotid IMT values were directly correlated to age (R = 0.484; *p* = 0.002), glycemia (R = 0.334; *p* = 0.03), triglycerides (R = 0.450; *p* = 0.004) and alkaline phosphatase (R = 0.372; *p* = 0.01) while, as reported above, an inverse correlation was found with CatK levels (R = –0.350; *p* = 0.02). In a multivariate model with IMT as the dependent variable, age (β = 0.433; *p* = 0.001), triglycerides (β = 0.294; *p* = 0.03) and CatK (β = –0.202; *p* = 0.05) remained significant predictors while the correlations with glycemia and alkaline phosphatase, found at univariate analysis, were lost. Such model accounted for 48% of the total variance of IMT (*p *< 0.0001). Correlations of IMT are resumed in Table 4 and Fig. 3.

**Table 4.** **Univariate and multiple regression analysis of mean carotid IMT values**.

	***Univariate correlation coefficient***	***p***
(log)Cathepsin-K	–0.350	0.02
Age	0.484	0.002
Glycemia	0.334	0.03
Triglycerides	0.450	0.004
Alkaline phosphatase	0.372	0.01
	***Multivariate standardized correlation coefficient (β)***	***p***
**Age**	**0.433**	**0.001**
**Triglycerides**	**0.294**	**0.03**
**(log)Cathepsin-K**	**–0.202**	**0.05**
Glycemia	–0.017	0.87
Alkaline phosphatase	0.221	0.16

Multiple R = 0.69, $R^{2}$ = 48%; *p *< 0.0001.

### 3.4 Subclinical Atherosclerosis in Ktx Recipients

**Fig. 4.**
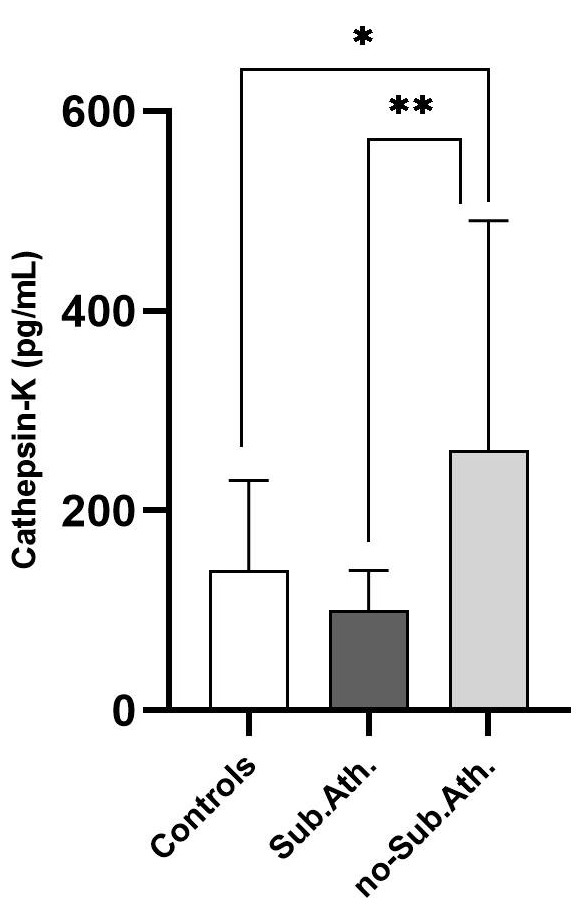
**Cathepsin-K levels in Ktx patients with (n = 17) or without (n = 23) subclinical atherosclerosis and in healthy controls (n = 30)**. *p = 0.01; **p = 0.002.

Seventeen patients (42.5%) showed evidence of subclinical atherosclerosis (mean IMT >0.9 and/or one unilateral IMT measurement above the 75th percentile of the age-gender reference range). These individuals had higher alkaline phosphatase (90.2 ± 19 vs. 74 ± 18.7 U/L; *p* = 0.008) and triglycerides levels (169.6 ± 63.4 vs. 120.6 ± 38.4 mg/dL; *p* = 0.003), a higher prevalence of diabetes (*p* = 0.04) and a lower E/e’ ratio (7.1 ± 1.2 vs. 11.5 ± 2.1; *p* = 0.04) as compared with those with normal IMT (Tables 1,2). No further differences were noticed with respect to the other parameters recorded, including ezetimibe treatment and the immunosuppressant regimen. CatK levels were apparently lower in Ktx patients with subclinical atherosclerosis as compared with healthy controls, although this difference did not attain statistical significance (100 [27.5–150] vs. 140 [50–240] pg/mL; *p* = 0.12). Conversely, CatK levels were higher in patients without evidence of subclinical atherosclerosis (260 [135–490] pg/mL) as compared with both healthy controls (*p* = 0.01) and patients with subclinical atherosclerosis (*p* = 0.002). Fig. 4 depicts differences in CatK among study subgroups.

At logistic regression analyses, alkaline phosphatase (OR 1.873; 95% CI 1.001–3.515; *p* = 0.05), triglycerides (OR 1.200; 95% CI 1.001–1.454; *p* = 0.05) and, above all, CatK (OR 0.761; 95% CI 0.569–0.918, *p* = 0.04) were confirmed as significantly associated with the presence of subclinical atherosclerosis while diabetes and E/e’ values were apparently not (Table 5). In such regard, CatK also displayed a remarkable diagnostic capacity in identifying Ktx patients with subclinical atherosclerosis, showing an area under the curve of 0.763 (95% CI 0.601–0.926; *p* = 0.001), with an optimal cut-off of 140 pg/mL holding a sensitivity of 76.47 (95% CI 50.1–93.2) and a specificity of 78.26 (95% CI 56.3–92.5).

**Table 5.** **Logistic regression analysis of clinical predictors of subclinical atherosclerosis. Statistically significant associations are highlighted in bold**.

	Unit of increase	OR	95% CI	*p*
**Cathepsin-K**	**100 pg/mL**	**0.761**	**0.569–0.918**	**0.04**
**Alkaline Phosphatase**	**10 U/L**	**1.873**	**1.001–3.515**	**0.05**
**Triglycerides**	**10 mg/dL**	**1.200**	**1.001–1.454**	**0.05**
Diabetes	Presence	8.145	0.449–147.879	0.15
E/e’	1 unit	1.107	0.968–1.265	0.21

## 4. Discussion

Findings from our study raises two main points for discussion. First, Cathepsin-K levels in Ktx recipients were lower as compared with individuals on maintenance hemodialysis (HD). No less important, such levels were almost comparable to those measured in healthy controls. Hence, Ktx seems to normalize the altered balance in CatK which characterizes HD patients [13, 14]. As previously observed, increased CatK in HD patients are largely influenced by the severity of bone mineral alterations and inflammation [14]. Ktx is known to ameliorate most of the complications related to advanced CKD. In particular, tangible benefits on systemic inflammation, oxidative stress and, above all, fluid and mineral metabolism become already evident few weeks after transplantation and persist over time as long as the implanted kidney continues working [21]. Indeed, our Ktx recipients displayed on average normal or nearly normal parathormone and inflammatory indexes and no independent correlations were found between CatK levels and such parameters. Conversely, a robust, inverse association was found between CatK levels and Ktx vintage; this would reinforce the hypothesis that a longer lasting recovery in renal function may play a crucial role also in normalizing systemic CatK release and activity.

CatK usually abounds in lysosome of osteoclasts and macrophages, which is in line with its well-established function of extracellular matrix remodelling [22].

Nevertheless, CatK also promotes leukocyte recruitment and elicits pro-inflammatory processes through cross-talk with the coagulation cascade [23, 24]. As CatK is upregulated in various systemic inflammatory and autoimmune diseases [25], a direct inhibitory effect of immunosuppressive therapy on circulating CatK levels in our Ktx cohort cannot be in principle excluded and would deserve appropriate investigation by targeted mechanistic studies. No less important, the strong independent correlation found with the left ventricle end-diastolic volume merits further examination as it suggests a biological involvement of CatK also in the cardiac morpho-functional adaptations which characterize renal patients. Such a hypothesis would pair well with recent studies demonstrating a direct role of cysteine proteases in pathological cardiac remodelling, particularly in chronic heart failure [26, 27].

Another important aspect of our study pertains the strong interplay found in Ktx recipients between CatK levels and subclinical atherosclerosis, as assessed by carotid IMT evaluation. Such observation is corroborated by various findings. First, we found a remarkable, inverse relationship between circulating CatK levels and mean IMT values. Of note, such an association remained independent from potential confounders in two different multivariate models employing, in turn, CatK and IMT as the dependent variable. Second, Ktx patients with evidence of incipient atherosclerosis—that is displaying a mean IMT >0.9 and/or one unilateral IMT measurement above the 75th percentile of the age-gender reference range- showed reduced CatK levels as compared to those without. Of note, these latter exhibited significantly higher CatK levels with respect to healthy subjects, while the study was apparently underpowered to catch differences between Ktx patients with pathological IMT and healthy individuals. No less important, logistic regression analyses confirmed that an increase in circulating CatK levels held an apparently beneficial meaning towards the presence of subclinical atherosclerosis. More in detail, in this cohort, an outstanding 24% reduction in the odds ratio of this complication was noticed for every 100 pg/mL increase in circulating CatK and exploratory ROC analyses showed a very interesting discriminatory capacity of this substance (AUC 0.763) to identify Ktx patients with pathological IMT. This latter finding is of particular interest, as it may candidate CatK as a new, promising tool for improving early diagnosis and risk stratification in individuals prone to develop atherosclerotic vascular disease.

We cannot clarify the exact biological meaning of the reduced circulating CatK levels found in the presence of incipient atherosclerosis. One possible explanation relies on a systemic down-regulation of this protein to compensate the early vascular damage and prevent disease progression towards plaque formation. It is widely acknowledged that CatK activity is essential for normal vascular tissue remodelling [28]. However, as elsewhere demonstrated [8], an enhanced CatK activity promotes instability and rupture of atherosclerotic plaques, while disrupting the CatK gene reduces plaque progression and induces fibrotic transition [29]. Accordingly, CatK levels in patients with overt coronary heart disease are positively correlated with plaque volume but inversely associated with the fibrotic content [11].

Unfortunately, to the best of our knowledge, no clinical or mechanistic studies have so far evaluated CatK in early atherosclerosis. Hence, this “first glance hypothesis” would need confirmation by focused studies modelled on such particular conditions. Immunosuppressant agents, particularly m-TOR inhibitors, are known to limit atherosclerosis progression in Ktx recipients by exerting a direct effect on immune cells at the vascular wall level [30]; by the same token, a similar inhibitory effect on CatK expression cannot in principle be excluded. Nevertheless, all study participants were under chronic immunosuppressive therapy and no differences in the rate of different medications prescribed were noticed between the two study subgroups.

Our study has some limitations that deserve mentioning. First, the sample size was relatively small, although enough powered to avoid overfitting of the statistical models. Larger studies in more heterogeneous cohorts with respect to type of Ktx, immunosuppressive regimen, residual renal function, severity of atherosclerosis and CV comorbidities are necessary to generalize our findings as selection bias cannot be fully excluded. Second, the lack of a longitudinal observation prevents to evaluate whether fluctuations in CatK over time pair with structural changes in IMT. This information would be crucial for refining the biological interpretation of the interplay between CatK and IMT, as well as for explaining whether a causal relationship exists between this factor and early vascular damage. In this view, we also acknowledge that the lack of additional instrumental information on vascular function and status (e.g., pulse wave velocity, carotid wall shear stress, plaque composition…) may limit findings interpretation: future studies encompassing a larger pattern of cardiovascular functional examinations in relationship with circulating CatK measurement would therefore be advocated.

## 5. Conclusions

In this study, we found decreased CatK levels in Ktx as compared to chronic HD patients. Future studies are needed to confirm the usefulness of CatK as a biomarker for early CV risk stratification and to clarify the exact pathophysiological mechanisms underlying the close relationships with the atherosclerotic process in the Ktx setting.
